# Re-Optimized Energy Levels and Ritz Wavelengths of ^198^Hg I

**DOI:** 10.6028/jres.116.008

**Published:** 2011-04-01

**Authors:** Alexander Kramida

**Affiliations:** National Institute of Standards and Technology, Gaithersburg, MD 20899

**Keywords:** atomic spectra, energy levels, mercury atom, reference data, standard wavelength

## Abstract

The procedure of level optimization for the ^198^Hg I spectrum emitted by electrodeless discharge lamps is revisited. Wavelength-measurement uncertainties and systematic shifts caused by interactions with the buffer gas and by calibration procedures are carefully analyzed and accounted for. Based on this extended analysis of the original measurements, the energy levels are re-optimized, which results in an improved set of level values consistent with the revised measured wavelengths. The newly obtained reliable Ritz wavelengths can be used as secondary wavelength standards in the wide range from 1200 Å to 65 000 Å.

## 1. Introduction

The nucleus of the isotope 198 of mercury has a zero nuclear magnetic moment. Thus, due to the absence of hyperfine structure, spectral lines of ^198^Hg in low-pressure discharges are very narrow and have long been used as a source of secondary wavelength standards (see, for example, Kaufman and Edlén [1] or Quinn [2]). For this purpose, it is important to know the energy levels and Ritz wavelength values based on them with high precision.

In a recent NIST compilation [3], the energy levels of ^198^Hg were determined from selected measured wavelengths of 105 spectral lines from 12 published papers [1, 4–14]. In this level optimization procedure, reciprocal squares of wavelength-measurement uncertainties were used as weights. Therefore, the assumed uncertainties of the wavelengths strongly affect the resulting level values. Examination of the data in Ref. [3] shows that 14 out of 105 observed wavelengths deviate from the Ritz values by more than three times the measurement uncertainty. Four of these large deviations exceed five times the measurement uncertainty. If the measurement uncertainties had been estimated correctly, such large deviations would have been statistically impossible for such a small data set. Therefore, we conclude that the energy levels and observed wavelengths are internally inconsistent in Ref. [3].

The purpose of the present work is to eliminate these inconsistencies and provide a set of reliable Ritz wavelengths suitable as secondary wavelength standards. For this purpose, it is necessary to analyze the wavelength-measurement uncertainties, remove systematic shifts from the published wavelength values, and re-optimize the energy levels.

## 2. Analysis of Measurement Uncertainties

### 2.1 Measurements of Salit et al. [4] and Veza et al. [5]

These papers were among the main sources of wavelengths included in Ref. [3].

Salit et al. [4] measured four lines of ^198^Hg I emitted by water-cooled electrodeless discharge lamps using a Fourier-transform spectrometer (FTS). The measurements were calibrated by a laser locked to a precisely known hyperfine transition in molecular iodine. The relative uncertainty of the calibration, ultimately tied to the primary ^133^Cs standard, was 6 × 10^−9^. The measurements were made with lamps having different pressures of Ar carrier gas between 33 Pa and 1330 Pa (0.25 Torr and 10 Torr). The lamps were excited in a microwave discharge at 2450 MHz. Microwave power delivered to the discharge was in the range 20 W to 40 W.

These measurements were extended by Veza et al. [5] who used the same basic method (the same set of water-cooled electrodeless discharge lamps and FTS). The wavenumber scale of the FTS was established by the four ^198^Hg I lines measured by Salit et al. [4]. Veza et al. reported 30 measured wavenumbers of ^198^Hg I (including the four lines previously reported by Salit et al. [4]). 10 of these wavenumbers were measured at one Ar pressure only, 33 Pa. The total measurement uncertainties were close to those achieved by Salit et al. [4] (0.00015 cm^−1^ to 0.0009 cm^−1^)^1^.

We note that the measurement uncertainties were reported by Salit et al. and Veza et al. at the 95 % level of confidence (corresponding to two standard deviations). For the purpose of our present level optimization procedure, we divided these uncertainty values by a factor of two to convert them to the 68 % level of confidence (or one standard deviation). This was not done in Ref. [3].

Most of the wavelengths used in Ref. [3] were measured with 198Hg discharge lamps with argon as a carrier gas at a pressure of about 33 Pa (0.25 Torr). This choice was necessitated by the measurement precision, which was generally better at this pressure, and by a greater number of measurements available at that pressure as compared to other pressures. Following this convention, we include in the level optimization the wavelengths from Salit et al. [4] and Veza et al. [5] measured at this pressure of argon.

### 2.2 Measurements of Burns and Adams [6]

In Ref. [3], 35 wavelengths in the range 2300 Å to 6910 Å were quoted from Burns and Adams [6]. These interferometric measurements were made in air with electrodeless discharge lamps excited by radio-frequency radiation. The lamps had an argon buffer gas pressure of 260 Pa to 400 Pa (2 Torr to 3 Torr). The measurements of Burns and Adams are commonly believed to be accurate to ± 0.0002 Å [1, 3, 7]. However, for the purpose of estimation of the energy level values at a low argon pressure of 33 Pa, the influence of pressure shifts has to be taken into account. Among lines investigated by Veza et al. [5] at various Ar pressures, there are 20 lines between 2893 Å and 5790 Å previously measured by Burns and Adams [6]. Veza et al. [5] found that the shifts between wavenumbers measured at Ar pressures of 400 Pa and 33 Pa can be as large as 0.0024 cm^−1^ (for the 4339 Å line). All lines were found to be shifted by higher argon pressure to longer wavelength [4, 5]. The average shift computed as the root of mean square of shifts for all these 20 lines is 0.0011 cm^−1^. Assuming a median pressure of 330 Pa for the measurements of Burns and Adams [6], we estimate an average shift for their measured wavenumbers to be 0.0009 cm^−1^, which corresponds to 0.00005 Å at 2302 Å and 0.0004 Å at 6907 Å.

**Fig. 1:**
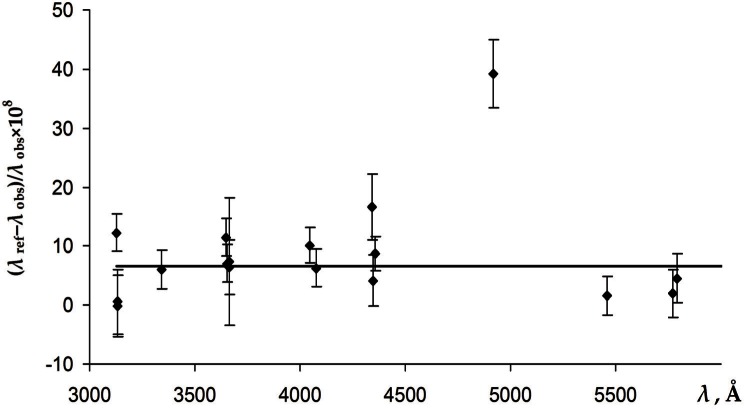
The differences from unity of the correction factors for the measurements of Burns and Adams [6] in the region 3125 Å to 5791 Å magnified by 10^8^. The error bars correspond to the assumed uncertainty of Burns and Adams (see text). The horizontal line corresponds to the average value of the correction factor equal to 1 + 65(12) × 10^−9^. The highly deviating point for the line at 4916 Å was excluded from the averaging. The plotted correction factors correspond to the argon pressure of 330 Pa.

Burns and Adams [6] used three different calibration standards in three wavelength regions, 3125 Å to 6709 Å, 2446 Å to 3125 Å, and 2260 Å to 2446 Å. The ^198^Hg line at 5460.7532 Å was used as standard for the long-wavelength region. Then the observed value 3125.6698 Å was used as standard for the medium-wavelength region. The observed wavelengths of the lines between 2576 Å and 2464 Å were used to evaluate the wavelengths below 2446 Å. The actual measured values in this type of interferometric measurements are ratios of observed and standard wavelengths. Therefore, if the standard wavelength used had a small error, a single multiplicative factor could be used to correct all wavelengths measured with the same calibration method. Since 17 of the 30 wavelengths listed by Burns and Adams in the range 3125 Å to 6709 Å were precisely measured by Salit et al. [4] and Veza et al. [5] at various argon pressures between 33 Pa and 1300 Pa, the values of these wavelengths can be linearly interpolated to the median pressure 330 Pa of Burns and Adams with high accuracy. Then a correction factor can be determined as an average of 17 individual estimates of these lines. This procedure is illustrated in Fig. 1, which depicts the differences of the correction factors for each line from unity, magnified by a factor of 10^8^.

Figure 1 shows that the measurements of Burns and Adams [6] in the region 3125 Å to 5791 Å, except for one highly deviating value for the line at 4916 Å, are consistent with a single correction factor equal to 1 + 65(12) × 10^−9^. The highly deviating measurement of the line at 4916 Å was excluded from the determination of the correction factor.

We corrected the wavelengths given by Burns and Adams [6] in the 3125 Å to 6908 Å range using the multiplicative correction factor given above. The corrected wavelengths are systematically longer than the original values. The correction is between 0.0002 Å and 0.0004 Å. We estimated the uncertainties of the corrected wavelengths as a sum in quadrature of the following quantities: 1) calibration uncertainty resulting from the uncertainty of the calibration factor (1.2 × 10^8^ times the observed wavelength), 2) statistical uncertainty (discussed below), 3) a contribution (amounting to ±0.00018 cm^−1^) due to the uncertainty of the argon pressure in [6], ±66 Pa (or ±0.5 Torr).

In the determination of the statistical uncertainties *δλ*_stat_, the main constituent *δλ*_var.c.f_ is due to the variance of the calibration factor and is equal to 4.5 × 10^8^ times the observed wavelength *λ.* In addition to that, the different numbers of measurements per line have to be taken into account. For each line listed in Table 1 of Burns and Adams [6] they indicated the number of measurements *N*_meas_ they used to derive the mean observed wavelength. Most of the wavelengths that we used to determine the calibration correction were determined from more than 30 measurements. The statistical uncertainty is the square root of the variance. The variance of the mean value is inversely proportional to *N*_meas_ – 1. For the lines involved in the derivation of the correction factor, the mean of 1/(*N*_meas_ – 1), which we denote as *w*_mean_, is approximately equal to 1/34. To determine the statistical uncertainties we weighted *δλ*_var.c.f_ by a factor equal to the square root of *w*_mean_/(*N*_meas_ – 1).

The resulting uncertainties, varying between ±0.00011 Å for the shortest wavelengths and ±0.0011 Å for the longest wavelengths, are pertinent to the wavelength values corresponding to the argon pressure of 330 Pa (2.5 Torr). For the purpose of the level optimization corresponding to the low argon pressure of 33 Pa (0.25 Torr), these uncertainties were combined in quadrature with the estimated average pressure shift between 330 Pa and 33 Pa, 0.0009 cm^−1^ (see above). The total combined uncertainties vary between ±0.00014 Å and ±0.0012 Å for the shortest and longest wavelengths, respectively.

**Fig. 2:**
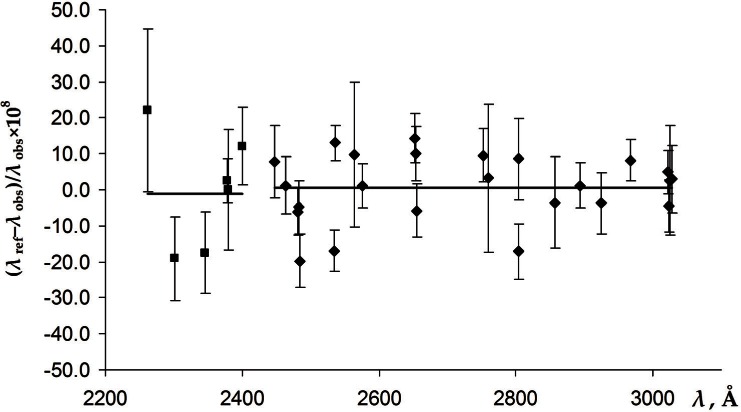
The differences from unity of the correction factors for the measurements of Burns and Adams [6] in the regions 2300 Å to 2440 Å (squares) and 2446 Å to 3028 Å (rhombs), magnified by 10^8^. The error bars correspond to the measurement uncertainties (see text) combined in quadrature with the uncertainties of the reference values. The horizontal lines correspond to the average values of the correction factors (see text).

In the region below 3125 Å, only four lines reported by Burns and Adams [6] were precisely measured by Veza et al. [5] at different pressures of argon. These are the lines at 3125.6698 Å, 3021.4996 Å, 2967.2832 Å, and 2893.5982 Å (air wavelengths from Burns and Adams [6]). The correction factors derived from these four lines strongly vary between 1 + 3.5 × 10^−8^ and 1 + 12 × 10^−8^. Therefore, re-calibrating the short-wavelength measurements of Burns and Adams using the same method as described above would be very unreliable. We chose a different method of evaluating the wavelengths in this region. Namely, we directly compared the wavelengths reported by Burns and Adams [6] with the Ritz wavelengths resulting from the final iteration of our level optimization (see Sec. 3), pertinent to the argon pressure of 33 Pa. There are 28 precise Ritz values in the region 2446 Å to 3028 Å, most of which involve energy levels determined from more than one combination. In the region 2300 Å to 2400 Å there are only five precise Ritz wavelengths available. One additional reference point is provided by the measurement of Reader and Sansonetti [15] for the Hg II line at 2262.2102(5) Å, for which Burns and Adams [6] reported the value of 2262.2097 Å. Fig. 2 depicts the differences from unity of the correction factors determined as ratios of the reference wavelengths (pertinent to argon pressure of 33 Pa) to the measurements of Burns and Adams [6]. For both short-wavelength regions, 2446 Å to 3028 Å and below 2446 Å, the average correction factors turned out to be very close to unity, 1 + 1(10) × 10^−8^, and 1 – 1(8) × 10^−8^, which means there is essentially no correction. Therefore, we left the measured wavelengths of Burns and Adams below 3028 Å unchanged.

The measurement uncertainties of the corrected wavelengths were estimated as the square root of the variance of the correction factor weighted by the square root of the ratio *w*_mean_/(*N*_meas_ – 1), where *w*_mean_ was determined separately for the two short-wavelength regions 2446 Å to 3028 Å and below 2446 Å and was found to be 12.6 and 7.4, respectively. In the region 2446 Å to 3028 Å, the uncertainties vary between ±0.00012 Å and ±0.0005 Å, depending mainly on the number of measurements of the line. Below 2446 × Å, the uncertainties are between ±0.00012 Å and ± 0.00026 Å. Since the Ritz wavelengths used in this derivation pertain to the argon pressure of 33 Å, these estimates already include the possible pressure shifts.

The wavelength values given by Burns and Adams [6] with only three digits after the decimal point were evaluated separately. On average, they differ from much more precise Ritz values by ±0.0025 Å. This average deviation was adopted as an estimate of the uncertainty of these wavelengths.

A special note should be made in connection with the lines from the 6s9d ^3^D_2_ level. In the line list of Burns and Adams [6] there are three such lines at 2399.3485 Å, 2699.378 Å, and 3701.4322 Å (our corrected wavelength of the latter line is 3701.4324(6) Å). The wavelength of the first of these lines was determined as a mean of 15 measurements [6], while for the other two lines there were only one and four observations, respectively. Therefore, the position of the 6s9d ^3^D_2_ level is mainly determined by the observed wavelength of the first line, 2399.34850(14) Å. The Ritz wavelength of the 2699.378 Å line is 2699.37548(18) Å, which marginally agrees with the measurement within its adopted uncertainty, ±0.0025 Å. However, for the line at 3701.4324 Å the Ritz wavelength, 3701.4367(3) Å deviates from the observed value by 0.0043 Å, which exceeds the estimated measurement uncertainty (±0.0006 Å) by a factor of 7.2. This large deviation suggests that there was some error in the four measurements of this line. The anomalously large isotope shift between ^202^Hg and ^198^Hg (0.004 Å [6]) is another indicator of an error. This line corresponds to the *n* = 9 member of the 6s6p ^1^P°_1_ – 6s*n*d ^3^D_2_ series. For the other members of the same series (*n* = 6, 7, and 8), the isotope shifts (^202^Hg – ^198^Hg) are 0.0016, 0.0007, and –0.0006 Å, respectively [6]. Based on these considerations, we excluded the 3701.4324 Å line from the level optimization procedure.

In addition to the calibration errors considered above, the measurements of Burns and Adams [6] could be affected by uncertainties in the refractive index of air. The wavelengths were measured in air and then converted to standard air (101.325 kPa, 15 °C) using the formulas for the refractive index of air from Meggers and Peters [16]. This formula is rather inaccurate and does not take into account the humidity and the contribution of CO_2_. The possible differences of the ambient conditions from standard would reveal themselves as a systematic deviation of the calibration factors depicted in Figs. 1 and 2 depending on the wavelength. However, these figures do not exhibit any noticeable variation of the correction factors with wavelength. Therefore, the influence of the air refraction on the measurements of Burns and Adams [6] can be neglected.

### 2.3 Measurements of Herzberg [7]

To excite the spectrum of ^198^Hg, Herzberg [7] used an electrodeless discharge through a mixture of ^198^Hg vapor and krypton. The krypton pressure was not specified in his paper. However, it was mentioned that this pressure was as low as compatible with the smooth running of the discharge. The spectrum was photographed with a 6.4 m normal-incidence spectrograph. Most of the lines were measured against iron lines in the same (second) order of diffraction. However, some of the red and infrared lines that were observed only in first order of diffraction were measured against Fe or Ne lines of second or third order. For lines measured in the same order as reference lines, Herzberg estimated the uncertainty as ±0.002 Å. For the lines above 8000 Å, his estimated uncertainty was ± 0.005 Å. In [3], the adopted uncertainty of Herzberg’s measurements was ±0.002 Å below 9500 Å and ±0.005 Å above that. Assignment of the uncertainty of ±0.002 Å to the line at 9425.915 Å was an error, as it contradicts Herzberg’s specification of ± 0.005 Å for this wavelength region.

By comparing the differences between Herzberg’s observed wavelengths and the Ritz values from the final iteration of our level optimization (see Sec. 3) we found that his measured wavelengths above 8000 Å (which were all measured against third-order standards) have a systematic shift of – 0.019(6) Å. When this shift is removed, the corrected wavelengths show a standard deviation of ± 0.013 Å from the Ritz values. We adopted this increased value as an estimate of uncertainties in this wavelength region.

Comparison of Herzberg’s wavelengths below 8000 Å with the Ritz values shows no systematic shift. The standard deviation of his observed wavelengths from the Ritz values is 0.0029 Å, which is somewhat greater than his own estimate of uncertainty for this wavelength region, ±0.002 Å. We adopted this increased value as an estimate of the uncertainties in this wavelength region.

### 2.4 Measurements of Barger and Kessler [10]

Barger and Kessler [10] interferometrically measured the vacuum wavelengths of the two ^198^Hg lines at 2537 Å and 3132 Å against the ^86^Kr primary standard at 6057 Å using a ^198^Hg atomic beam and an ^86^Kr hot-cathode lamp. The resulting vacuum wavelengths were 2537.26871(3) Å and 3132.74985(4) Å. They also measured the 3132 Å line against the 2537 Å line for which they assumed the value of 2537.268711 Å. This resulted in the value of 3132.749847(6) Å for the vacuum wavelength of the 3121 Å line. This line was re-measured by Veza et al. [5] at several pressures of argon. They determined the vacuum wavelength extrapolated to zero argon pressure to be 3132.749895(20) Å.

The reference ^86^Kr lamp in the experiment of Barger and Kessler contained a 1.9 % admixture of ^84^Kr. To account for the change of the reference wavelength caused by a small admixture of isotope ^84^Kr, Barger and Kessler modeled the shape of the emitted Kr line accounting for the effect of instrumental parameters such as reflectance and spacing of the interferometer plates. They found that the wavelength of the reference line as observed in their experiment was longer than the standard value, 6057.802106 Å, adopted at that time, by 0.000019 Å. Furthermore, they noted that this wavelength might be affected by Doppler and Stark shifts. The values of additional corrections due to these effects, estimated by different authors, as quoted by Barger and Kessler, varied between –0.000008 Å and +0.0000033 Å. These considerations show that the value of the wavelength in the experiment of Barger and Kessler was uncertain. Therefore, since there is an independent precise measurement of the 3132 Å line of ^198^Hg described above, we can use it as a reference line and deduce the wavelength of the 2537 Å line from it. This is a reversal of the procedure used by Barger and Kessler to determine the wavelength of the 3132 Å line relative to the 2537 Å line.

We multiplied the ratio of the wavelengths of the 2537 Å and 3132 Å lines measured by Barger and Kessler [10] (see their Table 1b) by the vacuum wavelength of the 3132 Å line measured by Veza et al. [5], 3132.749895 Å, and obtained the value 2537.268750(17) Å, which corresponds to the wavelength in standard air 2536.506625(17) Å.^2^ For the purpose of determination of the energy levels at an argon pressure of 33 Pa (0.25 Torr), we increased the wavelength by 5 μÅ following from the pressure shift rate of 20 μÅ/Torr [17]. The resulting adopted wavelength in standard air is 2536.506630(17) Å.

Schweitzer [18] independently measured the vacuum wavelength of the 2536 Å line using a Zeeman absorption filter. His result, 2537.26874(3) Å in vacuum or 2536.50662(3) in standard air, agrees well with our corrected value based on the measurements of Barger and Kessler.

### 2.5 Measurements of Humphreys and Paul [8, 9, 13, 14]

Humphreys and Paul measured the ^198^Hg I wavelengths emitted by an electrodeless discharge lamp with a Fabry-Perot interferometer. There is a rather vague indication in their 1958 report [8] that the lamps they used contained argon as a carrier gas at a pressure of about 270 Pa (2 Torr). Furthermore, in their 1965 report [13] there is a hint that the lamps they used might have been the same in all their measurements. Therefore, we assume that effects of the pressure shifts were approximately the same in all their experiments and correspond to the argon pressure of 270 Pa. Measurements of Refs. [8] and [9] were made with a Fabry-Perot interferometer. In the first work [8] they were made in air, while in the second work the interferometer was placed in a vacuum chamber.

In Ref. [8] the absolute scale for the wavelength measurements was based on several Ar I lines between 8400 Å and 10470 Å. Their adopted wavelengths were found to be consistent with each other to within 1 part in 10^7^. The same degree of consistency was found also for the line of ^198^Hg I at the air wavelength of 10139.793 Å. Therefore, we adopt the value of 10^−7^ as the relative statistical uncertainty of these measurements. The measured wavelengths of ^198^Hg were given in Table 4 of Humphreys and Paul [8]. In the text they noted that the values in this table must all be increased by 0.005 Å to account for the phase shift correction. This was not done in Ref. [3]. The validity of this correction is verified by comparison of the two wavelengths of Ref. [8], 11287.401 Å and 13570.566 Å (air wavelengths) re-measured by them later in Ref. [9]. The new values, 11287.4056 Å and 13570.5714 Å are indeed greater than the old ones by 0.005 Å on average. Thus, we applied this correction to all wavelengths from Table 4 of Ref. [8]. A further correction is made by multiplying the wavelengths by a correction factor calculated as a weighted mean of ratios of the Ritz wavelengths (from the final iteration of our level optimization; see Sec. 3) of the four lines below 14000 Å to their values from Ref. [8]. This correction factor was found to be 1 – 2(7) × 10^−8^. The total uncertainties of the final values were estimated as a sum in quadrature of the following three components: 1) statistical uncertainty equal to 10^−7^ times wavelength, 2) calibration uncertainty (due to the uncertainty of the calibration factor) equal to 7 × 10^−8^ times wavelength, 3) uncertainty due to possible pressure shifts between argon pressure of 270 Pa and 33 Pa (estimated as 0.0007 cm^−1^ in wavenumber). The total uncertainties of the wavelengths longer than 16900 Å, estimated in this way, are equal to ±0.003 Å.

In Ref. [9], Humphreys and Paul used the vacuum wavelength of the ^198^Hg line at 10142.572 Å as a standard. They measured vacuum wavelengths of five other lines of ^198^Hg between 11280 Å and 15300 Å relative to this standard with statistical uncertainties in the range 0.0008 Å to 0.0021 Å. At this level of precision, discrepancies between different formulas for the refractive index of air begin to be visible in the last digits of values converted to standard air. The values we obtained from the vacuum wavelengths of Humphreys and Paul [9] with the five-parameter formula of Peck and Reeder [19] are smaller than their own results by 0.0001 Å on average. The values given in Ref. [3] were obtained in the same way as ours, except that we applied the conversion formula to the exact values of the mean vacuum wavelengths (averages of several individual measurements given by Humphreys and Paul), whereas the values in Ref. [3] were obtained by applying the same conversion formula to the values of the mean vacuum wavelengths rounded to four places after the decimal point. Thus, for the vacuum wavelength 12074.9066 Å we obtained the value in standard air 12071.6031 Å, while Ref. [3] gives 12071.6030 Å.

Our level optimization (see Sec. 3) produced the Ritz value of 10142.5725(2) Å for the vacuum wavelength of the line used by Humphreys and Paul [9] as standard. The resulting calibration correction factor is 1 + 49(20) × 10^−9^. This increased the wavelengths by (0.0006 to 0.0007) Å. The uncertainties were calculated in the same way as for reference [8] (see above). Their values are in the range 0.0012 Å to 0.0023 Å. It was possible to remove the pressure shift from the line at 12071.6 Å using the pressure shift rate determined by Kaufman [17], 0.00031 Å/Torr. The wavelength of this line at an Ar pressure of 33 Pa (0.25 Torr) is shorter than at pressure of 400 Pa (2 Torr) by 0.00054 Å. We included this correction in our list of compiled lines.

The infrared measurements in the 3.2 μm to 4.0 μm region [13, 14] were made in air with a plane-grating spectrometer described in detail by Plyler et al. [20]. The latter authors noted that with this instrument sharp lines separated by 0.15 cm^−1^ could be partially resolved. This gives a hint that the measurement uncertainty could be a fraction of this value. A quantitative estimate can be obtained by analyzing the ^198^Hg energy level values given by Humphreys and Paul [13]. They assumed that the 5g levels should be completely unresolved in their experiment and obtained a value of 79783.85(2) cm^−1^ from four lines combining the 5g group with the 5f ^1^F°_3_, ^3^F°_2_, ^3^F°_3_, and ^3^F°_4_ levels. Using the values used by them for these 5f levels, one can obtain the standard deviation of individual estimates of the 5g group position, which is 0.049 cm^−1^. This produces the statistical uncertainty for the average value equal to 0.025 cm^−1^, in fair agreement with the estimated uncertainty of ± 0.02 cm^−1^ given by Humphreys and Paul [13]. Therefore, we estimate the uncertainty in the measured wavenumbers to be ±0.05 cm^−1^. This corresponds to ±0.8 Å in wavelength. For the wavelength 35217.5 Å (in air), which was given in Ref. [13] with one digit after the decimal point, we assume a twice greater uncertainty. These uncertainty estimates are much greater than the values adopted in Ref. [3], ±0.05 Å and ±0.5 Å for the wavelengths with two and one digits after the decimal point, respectively.

The vacuum wavelength 39292.56(8) Å given in Ref. [3] was derived from the wavenumber 2545.011 cm^−1^ reported by Humphreys and Paul in their preliminary report [14]. In the later report [13] they gave a more detailed description of the origin of this value. Namely, it was calculated from the known energy levels of ^198^Hg. Table 2 of the latter reference lists this line with intensity of 5000, and in the text they also indicated that this line was the strongest one in this region of the spectrum. We assume that the Ritz value of this wavelength was much more precise than the observed one. Thus, we assign to this wavelength the same uncertainty as for the neighboring strong lines, ±0.8 Å, round it to one place after the decimal point, and exclude it from the level optimization procedure. The Ritz value of the wavelength following from our level optimization is indeed much more precise than it could be measured. Its uncertainty is ±0.016 Å.

We note that the original report [13] quoted in the compilation [3] was translated in French and published in the same year in Journal de Physique. We give this additional citation as it is easier to find.

### 2.6 Measurements of Peck et al. [11]

Peck et al. [11] measured the lines of neutral argon and ^198^Hg emitted by electrodeless discharge lamps with a Michelson interferometer. Although they did not specify the pressure of argon in their lamps, they mentioned that the lamps were supplied by C. J. Humphreys. Therefore, it is reasonable to assume that argon pressure in those lamps was the same as in the experiments of Humphreys and Paul (see the previous section).

All wavelengths were measured in that work relative to the Ar I line at 9784.5020 Å (wavelength in standard air). Peck et al. noted that the quantities they actually measured were the ratios of the wavelengths to the wavelength of the above reference line of argon. Therefore, their reported wavelengths are proportional to the same calibration factor equal to the ratio of 9784.5020 Å to the actual wavelength of this Ar I line.

To determine the value of this calibration factor, we compared their reported wavelengths for Ar I with those given by Sansonetti and Andrew [21], Whaling et al. [22], and Norlén [23]. Before making these comparisons, we corrected the wavelengths given by Whaling et al. [22] and Norlén [23] as recommended by Sansonetti [24]. Namely, we decreased the wavenumbers of Whaling et al. [22] by 6.7 parts in 10^8^ and increased the wavenumbers of Norlén [23] by the same proportion. The average value of the calibration factor determined from 19 wavelength comparisons is 1 + 33(25) × 10^−9^. We corrected the air wavelengths of ^198^Hg I given by Peck et al. [11] by multiplying them by this factor and increased the uncertainties by combining them in quadrature with the fraction of 2.5 parts in 10^8^ of the wavelength (this fraction corresponds to the uncertainty of the calibration factor). The uncertainties were further increased by combining them with the possible pressure shifts at an argon pressure of 270 Pa relative to pressure of 33 Pa, which we estimated as 0.0007 cm^−1^ (see Sec. 2.2).

### 2.7 Measurements of Kaufman [17]

The wavelength 2752.7830(1) Å was quoted in Ref. [3] from the compilation of Kaufman and Edlén [1]. The value given in the latter paper had been taken from the earlier work of Kaufman [17] and rounded off to four digits after the decimal place. Its uncertainty was stated in Ref. [1] as 5 parts in 10^8^, amounting to ±0.00014 Å for this wavelength. We found that a somewhat more accurate value of this wavelength can be obtained by re-analyzing the original measurements of Kaufman [17].

In the latter work, the vacuum wavelengths of 27 lines of ^198^Hg I were measured relative to the ^86^Kr I line at 6057.802106 Å, by photographic Fabry-Perot interferometry. Those measurements were made with ^198^Hg water-cooled electrodeless discharge lamps containing argon at pressures 33 Pa, 400 Pa, and 1330 Pa (0.25 Torr, 3 Torr, and 10 Torr, respectively). The 33 Pa (0.25 Torr) lamp had the same length of about 10 cm and internal diameter of 5 mm as the higher-pressure lamps but had an additional attached gas reservoir, which increased its total volume to 30 cm^3^. All lamps were water-cooled with the temperature maintained between 6 °C and 9 °C.

**Fig. 3:**
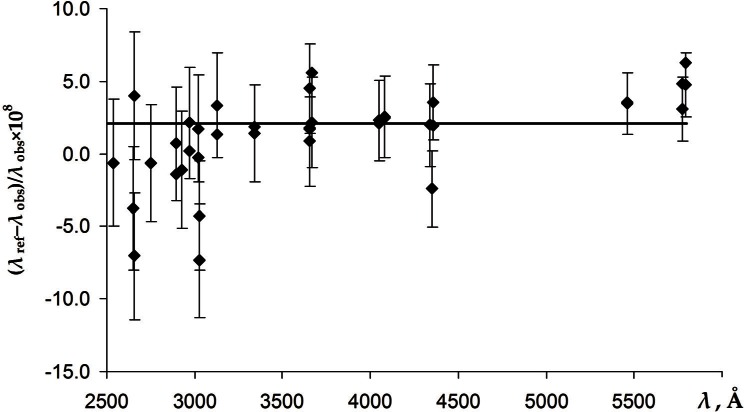
The differences from unity of the correction factors for the measurements of Kaufman [17] magnified by 10^8^. The error bars correspond to the sum in quadrature of the measurement uncertainties of Kaufman [17], ±0.00011 Å, and the reference wavelengths taken from Salit et al. [4], Veza et al. [5], and Barger and Kessler [10] (corrected as described in Sec. 2.4). The horizontal line corresponds to the weighted mean value of the correction factor equal to 1 + 21(5) × 10^−9^. The plot includes 23 data points from Kaufman’s measurements at an Ar pressure of 33 Pa and 14 points from the pressure of 400 Pa.

The ^86^Kr lamp used by Kaufman [17] for wavelength calibration was a capillary hot-cathode discharge lamp filled with 99.7 % ^86^Kr and 0.3 % ^84^Kr. It was operated within the conditions recommended by the International Committee of Weights and Measures. The emission of the reference 6057.802106 Å line was observed along the capillary in the direction cathode-to-anode-to-observer. Under these operational conditions, the observed wavelength of this line should have been affected by small Doppler and Stark shifts. Different estimates of the predicted net shift quoted by Kaufman [17] range between +4 μÅ and +15 μÅ. Kaufman made no correction for this shift because of the uncertainty of its value. Furthermore, measuring the ^198^Hg lines against the ^86^Kr standard, as it was done by Kaufman [17], might have introduced additional systematic shifts caused by different illumination of the interferometer by the different light sources. Since the ^198^Hg measurements of Salit et al. [4], Veza et al. [5], and Barger and Kessler [10] provide numerous high-precision internal standards, we can use them to re-calibrate the wavelength scale of Kaufman [17]. We did it in the same way as described for the measurements of Burns and Adams [6] (see Sec. 2.2). A scatter plot of the differences of the resulting correction factors from unity magnified by a factor of 10^8^ is depicted in Fig. 3.

The weighted mean value of the correction factor with weights inversely proportional to the square of the combined uncertainty is 1 + 21(5) × 10^−9^. We corrected the original wavelength measurements of Kaufman [17] by multiplying them by this factor and determined the average measurement uncertainty as a standard deviation of the resulting corrected wavelengths from the reference values. The wavelength of the 2752 Å line determined in this way is 2752.78314(11) Å.

### 2.8 Measurements of Davison et al. [25]

Davison et al. [25] used Fabry-Perot interferometry in vacuum to measure nine lines of ^198^Hg I emitted by an electrodeless discharge lamp filled with argon as a carrier gas at a pressure of about 33 Pa (0.25 Torr). They used these measurements to calibrate their measurements of the thorium spectrum. Among the ^198^Hg lines listed by them, there is a line at 2698.1500 Å (vacuum wavelength), which they used as a primary standard. The value of this wavelength was supplied to Davison et al. by Kessler in a private communication. In their Table 1 they also listed the wavelength of this line independently measured by Kaufman and privately communicated to them. Kaufman’s value was 2698.1499 Å. This line was not included in the compilation [3] because it was not identified with any possible transition between the known energy levels of ^198^Hg. The wavelength of this line in standard air is 2697.3497 Å. The line at an air wavelength of 2697.30(20) Å was observed by Murakawa [26] in the spectrum of natural mixture of isotopes of Hg and was identified by him as an unresolved blend of the forbidden electric-quadrupole transitions 6s6p ^3^P°_2_ – 6s7f ^3^F°_2,3,4_. A large number of similar forbidden transitions were observed by Murakawa and other researchers in arc spectra of mercury. They become allowed in the presence of a weak electric field, which mixes the states of different parities. The now-known Ritz wavelengths of the fine structure components of the blend at 2697.30 Å are 2697.25(2) Å, 2697.345(15) Å, and 2697.48(2) Å for the upper levels with *J* = 4, 3, and 2, respectively [27]. This allows us to unambiguously identify the line observed by Davison et al. as the 6s6p ^3^P°_2_ – 6s7f ^3^F°_3_ transition.

Comparison of the wavelengths of the other 8 lines of ^198^Hg measured by Davison et al. [25] with our much more precise Ritz values shows that their measurement uncertainties are ±0.00016 Å with an average correction factor of 1 + 14(18) × 10^−9^. The corrected air wavelength of this line is 2597.34970(16) Å. On the other hand, Kaufman’s wavelengths given in Table 1 of Davison et al. coincide with his published values [17] rounded off to four digits after the decimal point. Therefore, the same correction factor as determined in Sec. 2.7 applies to Kaufman’s value of the vacuum wavelength, 2698.1499 Å. The corrected air wavelength from Kaufman’s measurement is 2597.34959(11) Å, where the uncertainty does not account for the rounding error in the vacuum wavelength. The rounded-off value 2597.3496 Å coincides with the air wavelength calculated from the uncorrected vacuum value used by Davison et al. (which is in fact due to Kessler). We adopt this value with an increased uncertainty of ±0.0002 Å (accounting for the rounding error) and give a reference to Davison et al. [25] for simplicity.

### 2.9 Measurements of Baird et al. [12]

Baird et al. [12] measured the vacuum wavelengths of five lines of ^198^Hg I emitted by electrodeless discharge lamps using Fabry-Perot interferometry. The lamps used in that work were excited by radio-frequency radiation with a frequency of about 200 MHz. Most of the measurements were done with a lamp having a large reservoir containing about 3 mg of ^198^Hg and argon at about 33 Pa (0.25 Torr). The temperature of the lamp was maintained below 10 °C. All measurements were calibrated against the 6057 Å line of ^86^Kr. The Kr gas in the reference lamp was 99.5 % pure isotope ^86^Kr, and the isotope shift of the reference line was estimated to be smaller than 10 μÅ (10^−15^ m). The ^86^Kr lamps were operated as recommended by the International Committee of Weights and Measures (CIPM) in 1960. The Doppler shift in the reference lamp was eliminated by making measurements at alternating orientations of the lamp. The absence of Doppler shifts was verified by comparisons with measurements taken with ac discharges.

Four of the five ^198^Hg wavelengths measured by Baird et al. [12] were included in the compilation [3]. In the present work, we replaced these values with those measured by Salit et al. [4] and Veza et al. [5] because those newer values have smaller total uncertainties. However, it is interesting to compare the results of Baird et al. with the more precise Ritz values obtained in the present work (based mainly on the measurements of Veza et al.).

**Fig. 4:**
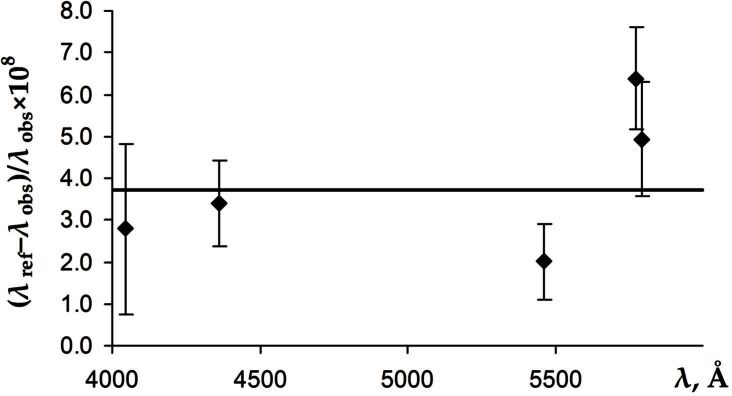
The differences of the calibration factors of the measurements of Baird et al. [12] from unity, magnified by 10^8^. The error bars represent the statistical uncertainties given by Baird et al. [12] combined in quadrature with the uncertainties of the Ritz wavelengths. The horizontal line corresponds to the weighted mean value of the correction factor equal to 1 + 37(5) × 10^−9^.

We assume that, similar to the Fabry-Perot measurements of Barger and Kessler [10] and Kaufman [17], the results of Baird et al. [12] may be affected by a calibration error. For each of the five ^198^Hg lines measured by them we compute the value of a calibration factor as a ratio of the Ritz wavelength to the one reported by Baird et al. The differences of those calibration factors from unity, expanded by a factor of 10^8^, are plotted in Fig. 4.

## 3. Level Optimization

The least-squares level optimization procedure was made with the computer code LOPT [28] in several iterations. In this procedure, the measured wavelengths weighted by reciprocal squares of their uncertainties are used to derive a set of energy level values that fits the observed wavelengths. In the first iteration, we used uncorrected original measured wavelengths with the authors’ uncertainty estimates from the sources described in Sec. 2. Then the original wavelength values from Burns and Adams [6], Herzberg [7], Barger and Kessler [10], Humphreys and Paul [8, 9], and Kaufman [17] were corrected by applying calibration corrections following from the Ritz wavelengths obtained in the level optimization, and the measurement uncertainty values were re-evaluated as described in Sec. 2. The process was repeated until convergence was reached (i.e., the Ritz wavelengths and their uncertainties, as well as the calibration corrections stopped changing after the level optimization).

The list of the resulting optimized energy levels is given in Table 1. Only one transition connecting excited levels to the ground level has been measured in ^198^Hg. It is the 6s^2 1^S_0_ – 6s6p ^3^P°_1_ transition at an air wavelength of 2536.506630(17) Å observed by Barger and Kessler [10] (see Sec. 2.4). Therefore, the level values are much better defined relative to the 6s6p ^3^P° levels than relative to the ground state. Among the 6s6p ^3^P° levels, the ^3^P°_2_ level has the greatest number of accurately measured lines. Therefore, in Table 1 we give for all levels the uncertainties corresponding to the uncertainty of their separation from the 6s6p ^3^P°_2_ level. To obtain the uncertainties relative to the ground state, the uncertainty values from Table 1 must be combined in quadrature with the uncertainty of the ground level, ±0.0003 cm^−1^.

The list of observed and Ritz wavelengths of ^198^Hg I is given in Table 2. We included in this table Ritz wavelengths of transitions that were not measured in ^198^Hg but were observed in the natural mixture of isotopes of Hg.

In total, there are 78 observed transitions between levels involved in more than one combination (excluding the line at 3701.4324 Å that was not included in the level optimization procedure). For 18 of these transitions (23 % of the total number), the deviations of observed wavelengths (adjusted as described in Sec. 2) from the Ritz values exceeded one value of the assumed wavelength-measurement uncertainty, and for three transitions these deviations exceeded two values of uncertainties. This small number of outlying measurements is consistent with statistical distribution expected for unbiased normally distributed measurements. This confirms that our procedure is internally consistent.

## 4. Discussion

The origin of the systematic shifts present in the ^198^Hg measurements of Kaufman [17], and Baird et al. [12] (see Sec. 2) is not understood. If the same calibration correction as derived from the ^198^Hg measurements is applied to the wavelength of the orange line of ^86^Kr, 6057.802106 Å, which those authors used as standard, it would imply that this line was shifted in their experiments by + 128(36) μÅ and + 224(30) μÅ, respectively. The relative shifts would be 2.1(6) and 3.7(5) parts in 10^8^, respectively. Such large errors in the value of the ^86^Kr standard wavelength can be ruled out because reproducibility of this wavelength was established to be better than 1 part in 10^8^ by many investigators before 1960, when this wavelength was adopted as standard by CIPM (see Baird and Howlett [29]). The operational conditions of the ^86^Kr lamp were well defined in the standard specifications and were relatively easy to implement. Therefore, we can conclude that the source of the systematic shifts is related to the ^198^Hg measurements and not ^86^Kr.

One possible cause of the shifts is associated with the phase shifts in the Fabry-Perot interferometers. These shifts are caused by penetration of the light into the reflecting material covering the surfaces of the interferometer. This penetration changes the effective distance between the interferometer plates. The errors due to the imprecise knowledge of the phase shifts are usually eliminated by taking measurements with several etalon lengths. Furthermore, such errors are minimal at wavelengths close to the reference line and increase for greater wavelength separation. No such trend is noticeable in the measurements of Baird et al. (see Fig. 4). Such trend may be present in the measurements of Kaufman [17] and Burns and Adams [6] at the shortest wavelengths (see Figs. 3 and 2). However, statistical uncertainties are too large to draw a definite conclusion.

Another possible explanation of discrepancies in the ^198^Hg wavelengths could be the presence of uncontrolled Stark shifts. Such shifts can be caused by ambient electric fields that were not measured or controlled in the operation of the electrodeless discharge lamps. Generally, the radio-frequency or microwave radiation used to excite the discharge was maintained at minimum power required to sustain the discharge. However, the fact that the forbidden electric-quadrupole 6s6p ^3^P°_2_ – 6s7f ^3^F°_3_ transition at 2597.3496 Å was observed in several rf-excited lamps [25] gives evidence of the electric fields in these lamps being strong enough to permit this transition. On the other hand, this transition has the highest excited level (81106 cm^−1^) among those observed by Davison et al. [25]. The other transitions reported in that work have upper levels in the range 39412 – 77108 cm^−1^ and belong to the 6s6p, 6s6d, 6s7d, and 6s8d configurations. For these levels, especially the low-lying 6s6p ^3^P°_1_ level, Stark shifts should be much smaller than for 6s7f ^3^F°_3_. If the 6s6p ^3^P°_2_ – 6s7f ^3^F°_3_ transition, which Davison et al. used as reference, were significantly shifted by Stark effect, it would affect the wavelengths of all other measured lines, and would manifest itself as a systematic shift of all measured wavelengths in the same direction. We found no such systematic shift (or, at least, it is smaller than the measurement uncertainty). Therefore, Stark shifts are not likely to be the cause of the discrepancies.

The ^198^Hg lamps used by Burns et al. [6], Kaufman [17], Baird et al. [12], Salit et al. [4], Veza et al. [5] were basically all of the same type – sealed glass tubes filled with argon containing some ^198^Hg. The only significant difference between different investigations was in the excitation of the discharge. In the earlier works [6, 12] the lamps were excited by radio-frequency radiation, while Salit et al. [4] and Veza et al. [5] used microwave excitation.^3^ However, there are significant discrepancies in measured wavelengths even among the authors who used the same radio-frequency excitation of the discharge at the same argon pressure. These variations led CIPM [2] to adopt rather large uncertainties (on the order of 10^−8^) for the currently recommended values of the ^198^Hg wavelengths. For example, the measurements of Terrien [30], when reduced to the argon pressure of 33 Pa (0.25 Torr)^4^ using the known pressure shift rates from Veza et al. [5] deviate from our Ritz wavelengths by –12(6) parts in 10^9^ on average, while this deviation amounts to + 61(3) × 10^−9^ for the measurements of Bruce and Hill [31], + 37(6) × 10^−9^ for Baird et al. [12], and + 21(6) × 10^−9^ for Kaufman [17].

Another possible explanation of the wavelength shifts is the uncertainty of the argon pressure in the sealed lamps. In the process of manufacturing those lamps, the argon pressure is controlled before sealing. However, the sealing process heats the lamp at one point. After sealing, the pressure inside the lamp can no longer be measured. Terrien [30] noted, referring to Baird and Smith [32], that the pressure in the lamps after sealing might be significantly lower than the nominal value measured before sealing. He says that for the nominal 400 Pa (3 Torr) lamps the actual argon pressure may go down to (200 to 270) Pa (1.5 Torr to 2 Torr) after sealing. However, the pressure shift rates were measured by Baird and Smith [32] not with sealed lamps but with specially designed lamps with strictly controlled buffer gas pressure. The values of pressure shifts reported by Baird and Smith [32] on average agree well with the results of Veza et al. [5] obtained with sealed lamps. The weighted mean ratio of pressure shifts reported by Veza et al. [5] to those reported by Baird and Smith [32] is 1.10(10). This shows that the pressure in the sealed lamps (at least in the lamps used by Veza et al. [5] and Salit et al. [4]) is close to the nominal value before sealing. Moreover, the 33 Pa (0.25 Torr) lamps used by Baird et al. [12], Kaufman [17], Salit et al. [4], and Veza et al. [5] all had attached to them a large reservoir filled with argon. This reservoir greatly reduces the pressure changes caused by local heating of the glass in the process of sealing. It also reduces possible changes in the pressure due to local heating of the discharge by the exciting radiation. Therefore, uncertainty in the argon pressure can be ruled out from the list of possible causes of wavelength discrepancies.

We believe that the main cause of the discrepancies between the wavelengths reported by different authors is associated with the lamps themselves. However, its exact nature is still unknown. This problem should be investigated more thoroughly.

We note that Sansonetti and Veza [33] have recently measured the vacuum wavenumber of the 5460 Å line of ^198^Hg by using Doppler-free saturated absorption and frequency modulation laser spectroscopy. In that work, the mercury spectrum was observed in an outgassed and evacuated glass cell containing mercury. The measured wavenumber of this line, scaled to the argon buffer gas pressure of 33 Pa, is 18307.404950(16) cm^−1^. This value agrees well with our Ritz value, 18307.40491(11) cm^−1^. This proves that the scale of our wavelength values reported in Table 2 is accurate to within 6 parts in 10^9^. However, the values given in our tables should be used with caution, keeping in mind the yet unexplained possible variations of wavelengths emitted by different sealed lamps. These variations, as discussed above, can be as large as 6 parts in 10^8^.

Since the reason for the discrepancies between wavelengths measured by Veza et al. [5], Baird et al. [12], Kaufman [17], and Burns and Adams [6] is not known, the validity of the multiplicative corrections of the wavelength scale introduced in section 2 may be questioned. However, these correction factors were based on a number of wavelengths covering large ranges, and the statistical uncertainties of individual measurements are large enough to make the use of such multiplicative corrections reasonable. Nevertheless, at least some of the wavelengths we used as input to our least-squares level optimization procedure are known to contain systematic shifts. For example, the measurements of Burns and Adams were made at higher argon pressures than the majority of the other measurements we used. Therefore, they contain unknown pressure shifts, which affect the resulting Ritz values. The systematic errors in the Ritz wavelengths may be comparable in size with the given uncertainties of these values.

**Fig. 5:**
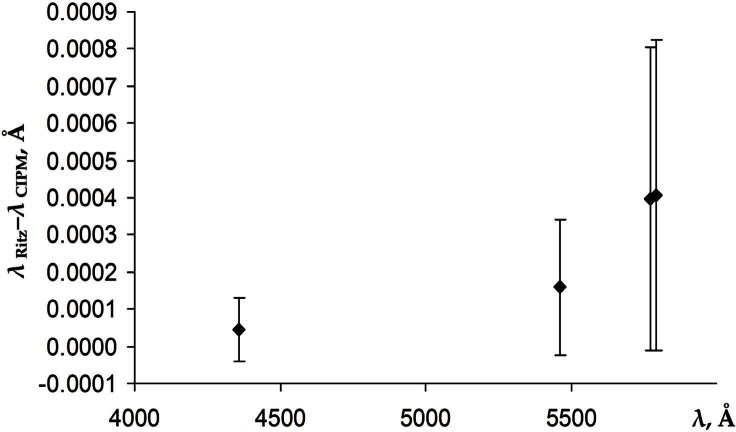
Comparison of ^198^Hg vacuum wavelengths recommended by CIPM [2] with the present Ritz values scaled to the argon pressure of 100 Pa.

It is interesting to compare our results with current CIPM recommendations [2], which remain unchanged since 1963. In Fig. 5 we compare the ^198^Hg wavelengths recommended by CIPM for electrodeless discharge lamps with 66 Pa to 133 Pa argon buffer gas with our Ritz values scaled to argon pressure of 100 Pa using the pressure shift rates from Veza et al. [5].

The error bars in Fig. 5 represent the uncertainties of the CIPM recommendations (reduced to the level of one standard deviation) combined in quadrature with the total uncertainties of our values. The latter are dominated by the uncertainties of the Ritz values given in Table 2 and include the uncertainties of the pressure shift rates from Veza et al. [5] and the breadth of the pressure interval in the CIPM recommendations (± 33 Pa). Although our values are in marginal agreement with the CIPM recommendations, there is a noticeable systematic difference. Our values are all greater than those of CIPM, and the difference increases with wavelength.

In this regard we note that presentation of the recommended wavelengths of atomic radiations in Sec. 2.2 of Quinn [2] is inadequate. The wavelength values, given in units of pm, are rounded to two digits after the decimal point, i.e., they are rounded to the nearest 0.01 pm. Their uncertainties are expressed in terms of “relative expanded uncertainty” *U*, which is defined in this case as three times the combined standard uncertainty *u_c_*. For ^86^Kr, *U* is given as 2 × 10^−8^, and for ^198^Hg, *U* = 5 × 10^−8^. Thus, the wavelength uncertainties on the level of 3 standard deviations are in the range (0.009 to 0.013) pm for ^86^Kr and (0.022 to 0.029) pm for ^198^Hg. If the wavelength values were not rounded so harshly, we could say that the standard uncertainties are three times smaller, i.e. (0.003 to 0.004) pm for ^86^Kr and (0.007 to 0.010) pm for ^198^Hg. However, rounding to the nearest 0.01 pm has drastically changed the statistical properties of the wavelength values and introduced some non-random errors depending on the actual values of the dropped digits. As the present author explained earlier [28], such rounded values as given in the CIPM recommendations [2] do not have a 99 % confidence level as would be true for values rounded less harshly (e.g., to the nearest 0.001 pm). For the ^86^Kr and ^198^Hg values, the rounding has decreased the confidence level to approximately (88 to 95) % and 98 %, respectively. This is roughly equivalent to an increase in the standard uncertainty values by 0.003 pm for ^86^Kr and by 0.001 pm to 0.002 pm for ^198^Hg.

## 5. Conclusion

In the present work, uncertainties and systematic errors in the original measurements of ^198^Hg wavelengths have been analyzed. The wavelengths reported in several sources have been adjusted to remove systematic shifts. The resulting set of wavelengths has been used in a least-squares level optimization procedure that produced a set of energy levels and Ritz wavelengths internally consistent with the evaluated measurements. Ritz wavelengths and their uncertainties have been obtained for lines in the range 1203 Å to 65000 Å. For 30 lines in the range 1849 Å to 5791 A the relative uncertainties are smaller than 10^−8^. These high-precision values correspond to the spectrum of ^198^Hg emitted by electrodeless discharge lamps filled with argon buffer gas at a pressure of 33 Pa (0.25 Torr). They are shifted from the values corresponding to the unperturbed ^198^Hg atom by up to 9 × 10^−9^ times wavenumber. Other (not completely known) perturbing effects present in sealed electrodeless discharge lamps may cause relative shifts in wavelengths as large as 6 × 10^−8^.

In comparison with Ref. [3], the new optimized energy level values are more consistent with the observed wavelengths. The values of 11 energy levels have been significantly changed (we consider the change as significant if the new value differs from the old one by more than 2 times the uncertainty given in Ref. [3]). One new energy level, 6s7f ^3^F°_3_, has been found.

## Figures and Tables

**Table 1:** Optimized energy levels of ^198^Hg I derived from observed spectral lines

Configuration	Term	*J*	Energy^a^ (cm^−1^)	Unc.^b^ (cm^−1^)	*N*_lines_^c^
5d^10^6s^2^	^1^S	0	0.0000	0.0003	1
5d^10^6s6p	^3^P°	0	37645.24175	0.00017	8
5d^10^6s6p	^3^P°	1	39412.45870	0.00012	20
5d^10^6s6p	^3^P°	2	44043.13571	–	16
5d^10^6s6p	^1^P°	1	54068.90351	0.00016	11
5d^10^6s7s	^3^S	1	62350.54062	0.00011	14
5d^10^6s7s	^1^S	0	63928.33517	0.0002	10
5d^9^6s^2^(^2^D_5/2_)6p	^2^[3/2]°	2	68886.4221	0.0010	2
5d^10^6s7p	^3^P°	1	69662.0129	0.0010	4
5d^10^6s7p	^3^P°	2	71207.54595	0.0007	1
5d^10^6s7p	^1^P°	1	71295.2065	0.0013	3
5d^10^6s6d	^1^D	2	71333.29605	0.00015	3
5d^10^6s6d	^3^D	1	71336.26217	0.00018	3
5d^10^6s6d	^3^D	2	71396.3265	0.00015	3
5d^10^6s6d	^3^D	3	71431.41544	0.00017	2
5d^10^6s8s	^3^S	1	73961.37870	0.00017	3
5d^10^6s8s	^1^S	0	74404.6797	0.0003	2
5d^10^6s8p	^3^P°	1	76467.153	0.006	1
5d^10^6s8p	^3^P°	2	76823.6461	0.0017	1
5d^10^6s8p	^1^P°	1	76863.336	0.004	2
5d^9^6s^2^(^2^D_5/2_)6p	^2^[7/2]°	4	76945.11	0.14	1
5d^10^6s7d	^1^D	2	77064.1870	0.00020	3
5d^10^6s7d	^3^D	1	77084.7218	0.0005	3
5d^10^6s7d	^3^D	2	77108.0180	0.00020	3
5d^10^6s7d	^3^D	3	77129.6465	0.0003	1
5d^10^6s5f	^3^F°	2	77236.9744	0.0010	1
5d^10^6s5f	^3^F°	3	77239.3008	0.0010	1
5d^10^6s5f	^1^F°	3	77241.6158	0.0010	1
5d^10^6s5f	^3^F°	4	77286.9746	0.0010	1
5d^10^6s9s	^3^S	1	78216.3382	0.0005	3
5d^10^6s9s	^1^S	0	78404.441	0.0020	2
5d^9^6s^2^(^2^D_5/2_)6p	^2^[3/2]°	1	78813.0887	0.0018	2
5d^10^6s9p	^3^P°	1	79412.857	0.008	1
5d^10^6s8d	^1^D	2	79660.8580	0.0005	4
5d^10^6s8d	^3^D	1	79678.8196	0.0016	3
5d^10^6s8d	^3^D	2	79690.3995	0.0014	4
5d^10^6s8d	^3^D	3	79702.7064	0.0004	1
5d^10^6s5g	^3^G	3	79783.746	0.05	1
5d^10^6s5g	^3^G	4	79783.78	0.05	1
5d^10^6s5g	^3^G	5	79783.83	0.05	1
5d^10^6s5g	^1^G	4	79783.855	0.05	1
5d^10^6s9p	^1^P°	1	79963.9315	0.0015	2
5d^10^6s10s	^3^S	1	80268.118	0.002	4
5d^10^6s10s	^1^S	0	80365.7344	0.002	1
5d^10^6s10p	^3^P°	1	80916.808	0.010	1
5d^10^6s10p	^3^P°	2	81022.870	0.009	1
5d^10^6s9d	^1^D	2	81057.825	0.002	3
5d^10^6s9d	^3^D	1	81071.138	0.003	2
5d^10^6s9d	^3^D	2	81077.754	0.002	2
5d^10^6s9d	^3^D	3	81085.220	0.004	1
5d^10^6s7f	^3^F°	3	81105.5678	0.002	1
5d^10^6s10p	^1^P°	1	81153.717	0.007	2
5d^10^6s11s	^3^S	1	81416.380	0.005	2
5d^10^6s11p	^3^P°	1	81808.211	0.011	1
5d^10^6s10d	^3^D	3	81913.65	0.04	1
5d^10^6s11p	^1^P°	1	81942.542	0.007	2
5d^10^6s12p	^3^P°	1	82378.867	0.012	1
5d^10^6s12p	^1^P°	1	82464.170	0.008	2
5d^10^6s13p	^3^P°	1	82766.085	0.012	1
5d^10^6s13p	^1^P°	1	82824.068	0.008	2
5d^10^6s14p	^1^P°	1	83082.187	0.012	1

a The energy values are applicable to electrodeless discharge lamps with Ar buffer gas pressure of 33 Pa.

b The uncertainties are given for the separation of the levels from 6s6p ^3^P°_2_. To obtain the uncertainties relative to the ground state, these values must be combined in quadrature with the uncertainty of the ground level, ±0.0003 cm^−1^.

c Number of observed transitions from which the level value is determined.

**Table 2:** Observed and Ritz wavelengths of ^198^Hg 1

*I*_obs_^a^ (acrb. u.)	*λ*_nbs_^b^ (Å)	*λ*_Ritz_^c^ (Å)	Δ*λ*_nbs-Ritz_ (Å)	*σ*_Ritz_ (cm^−1^)		Transition		Ref.
		1203.62744(18)		83082.187(12)	5d^10^6s^2^	^1^S_0_ – 5d^10^6s14p	^1^P°_1_	
		1207.37851(12)		82824.068(8)	5d^10^6s^2^	^1^S_0_ – 5d^10^6s13p	^1^P°_1_	
		1208.22436(18)		82766.085(12)	5d^10^6s^2^	^1^S_0_ – 5d^10^6s13p	^3^P°_1_	
		1212.64787(11)		82464.170(8)	5d^10^6s^2^	^1^S_0_ – 5d^10^6s12p	^1^P°_1_	
		1213.90356(17)		82378.867(12)	5d^10^6s^2^	^1^S_0_ – 5d^10^6s12p	^3^P°_1_	
		1220.36732(11)		81942.542(7)	5d^10^6s^2^	^1^S_0_ – 5d^10^6s11p	^1^P°_1_	
		1222.37119(16)		81808.211(11)	5d^10^6s^2^	^1^S_0_ – 5d^10^6s11p	^3^P°_1_	
		1232.22945(10)		81153.717(7)	5d^10^6s^2^	^1^S_0_ – 5d^10^6s10p	^1^P°_1_	
		1235.83718(15)		80916.808(10)	5d^10^6s^2^	^1^S_0_ – 5d^10^6s10p	^3^P°_1_	
200		1250.56382(2)		79963.9315(15)	5d^10^6s^2^	^1^S_0_ – 5d^10^6s9p	^1^P°_1_	
11		1259.24194(13)		79412.857(8)	5d^10^6s^2^	^1^S_0_ – 5d^10^6s9p	^3^P°_1_	
300		1268.82478(3)		78813.0887(19)	5d^10^6s^2^	^1^S_0_ – 5d^9^6s^2^(^2^D_5/2_)6p	^2^[3/2]°_1_	
		1301.01041(6)		76863.336(4)	5d^10^6s^2^	^1^S_0_ – 5d^10^6s8p	^1^P°_1_	
6		1307.75105(10)		76467.153(6)	5d^10^6s^2^	^1^S_0_ – 5d^10^6s8p	^3^P°_1_	
30		1402.61884(3)		71295.2065(13)	5d^10^6s^2^	^1^S_0_ – 5d^10^6s7p	^1^P°_1_	
14		1435.50259(2)		69662.0129(10)	5d^10^6s^2^	^1^S_0_ – 5d^10^6s7p	^3^P°_1_	
5000		1849.491917(11)		54068.9035(3)	5d^10^6s^2^	^1^S_0_ – 5d^10^6s6p	^1^P°_1_	
		2283.9054(2)		43771.138(5)	5d^10^6s6p	^3^P°_0_ – 5d^10^6s11s	^3^S_1_	
30	2302.0651(2)	2302.06466(14)	0.0004	43425.896(3)	5d^10^6s6p	^3^P°_0_ – 5d^10^6s9d	^3^D_1_	[6]
200	2345.4400(2)	2345.43959(13)	0.0004	42622.876(2)	5d^10^6s6p	^3^P°_0_ – 5d^10^6s10s	^3^S_1_	[6]
4000	2378.32460(12)	2378.32466(9)	−0.00006	42033.5779(16)	5d^10^6s6p	^3^P°_0_ – 5d^10^6s8d	^3^D_1_	[6]
30	2380.0040(3)	2380.0040(3)	0.0000	42003.921(5)	5d^10^6s6p	^3^P°_1_ – 5d^10^6s11s	^3^S_1_	[6]
		2397.74776(13)		41693.109(2)	5d^10^6s6p	^3^P°_1_ – 5d^10^6s7f	^3^F°_3_	
70	2399.34850(14)	2399.34851(14)	−0.00001	41665.295(2)	5d^10^6s6p	^3^P°_1_ – 5d^10^6s9d	^3^D_2_	[6]
30	2399.7293(2)	2399.72959(16)	−0.0003	41658.679(3)	5d^10^6s6p	^3^P°_1_ – 5d^10^6s9d	^3^D_1_	[6]
14	2400.497(3)	2400.49679(13)	0.000	41645.366(2)	5d^10^6s6p	^3^P°_1_ – 5d^10^6s9d	^1^D_2_	[6]
6		2441.06712(15)		40953.276(3)	5d^10^6s6p	^3^P°_1_ – 5d^10^6s10s	^1^S_0_	
30	2446.8998(2)	2446.89999(14)	−0.0002	40855.659(2)	5d^10^6s6p	^3^P°_1_ – 5d^10^6s10s	^3^S_1_	[6]
20	2464.06360(19)	2464.06363(3)	−0.00003	40571.0964(5)	5d^10^6s6p	^3^P°_0_ – 5d^10^6s9s	^3^S_1_	[6]
300	2481.99930(13)	2481.99915(9)	0.00015	40277.9408(14)	5d^10^6s6p	^3^P°_1_ – 5d^10^6s8d	^3^D_2_	[6]
90	2482.71310(16)	2482.71298(10)	0.00012	40266.3609(16)	5d^10^6s6p	^3^P°_1_ – 5d^10^6s8d	^3^D_1_	[6]
170	2483.82150(18)	2483.82101(3)	0.00049	40248.3993(5)	5d^10^6s6p	^3^P°_1_ – 5d^10^6s8d	^1^D_2_	[6]
2000	2534.76855(6)	2534.76867(3)	−0.00012	39439.4800(5)	5d^10^6s6p	^3^P°_0_ – 5d^10^6s7d	^3^D_1_	[5]
900000	2536.506630(17)			39412.4587(3)	5d^10^6s^2^	^1^S_0_ – 5d^10^6s6p	^3^P°_1_	[10]^d^
40	2563.8610(5)	2563.86125(13)	−0.0003	38991.9823(20)	5d^10^6s6p	^3^P°_1_ – 5d^10^6s9s	^1^S_0_	[6]
100	2576.29040(16)	2576.29043(3)	−0.00003	38803.8795(5)	5d^10^6s6p	^3^P°_1_ –5d^10^6s9s	^3^S_1_	[6]
6	2639.790(3)			37870.51(4)	5d^10^6s6p	^3^P°_2_ – 5d^10^6s10d	^3^D_3_	[6]
1600	2652.042836(25)	2652.042879(14)	−0.00004	37695.5593(2)	5d^10^6s6p	^3^P°_1_ – 5d^10^6s7d	^3^D_2_	[5]
6000	2653.68302(4)	2653.68297(3)	0.00005	37672.2631(5)	5d^10^6s6p	^3^P°_1_ – 5d^10^6s7d	^3^D_1_	[5]
400	2655.13029(4)	2655.130346(14)	−0.00006	37651.7283(2)	5d^10^6s6p	^3^P°_1_ – 5d^10^6s7d	^1^D_2_	[5]
6	2674.917(3)	2674.9160(3)	0.001	37373.244(5)	5d^10^6s6p	^3^P°_2_ – 5d^10^6s11s	^3^S_1_	[6]
	2697.34960(16)			37062.432(2)	5d^10^6s6p	^3^P°_2_ – 5d^10^6s7f	^3^F°_3_	[25]
200	2698.8314(3)			37042.084(4)	5d^10^6s6p	^3^P°_2_ – 5d^10^6s9d	^3^D_3_	[6]
100	2699.378(3)	2699.37548(12)	0.003	37034.618(2)	5d^10^6s6p	^3^P°_2_ – 5d^10^6s9d	^3^D_2_	[6]
		2699.85782(20)		37028.002(3)	5d^10^6s6p	^3^P°_2_ – 5d^10^6s9d	^3^D_1_	
		2700.82892(17)		37014.689(2)	5d^10^6s6p	^3^P°_2_ – 5d^10^6s9d	^1^D_2_	
400	2752.78314(11)	2752.783065(17)	0.00008	36316.1369(2)	5d^10^6s6p	^3^P°_0_ – 5d^10^6s8s	^3^S_1_	[17]^d^
10	2759.7103(5)	2759.71039(18)	−0.0001	36224.982(2)	5d^10^6s6p	^3^P°_2_ – 5d^10^6s10s	^3^S_1_	[6]
180	2803.47012(3)			35659.5707(4)	5d^10^6s6p	^3^P°_2_ – 5d^10^6s8d	^3^D_3_	[5]
40	2804.4378(3)	2804.43804(11)	−0.0002	35647.2638(14)	5d^10^6s6p	^3^P°_2_ – 5d^10^6s8d	^3^D_2_	[6]
3	2805.347(3)	2805.34940(13)	−0.002	35635.6839(16)	5d^10^6s6p	^3^P°_2_ – 5d^10^6s8d	^3^D_1_	[6]
3	2806.765(3)	2806.76417(4)	0.001	35617.7223(5)	5d^10^6s6p	^3^P°_2_ – 5d^10^6s8d	^1^D_2_	[6]
20	2856.9389(4)	2856.93880(2)	0.0001	34992.2210(3)	5d^10^6s6p	^3^P°_1_ – 5d^10^6s8s	^1^S_0_	[6]
800	2893.598253(25)	2893.598236(15)	0.00002	34548.92000(18)	5d^10^6s6p	^3^P°_1_ – 5d^10^6s8s	^3^S_1_	[5]
70	2925.41339(4)	2925.41339(4)	0.00000	34173.2025(5)	5d^10^6s6p	^3^P°_2_ – 5d^10^6s9s	^3^S_1_	[5]
3000	2967.283453(26)	2967.283442(17)	0.00001	33691.02042(20)	5d^10^6s6p	^3^P°_0_ – 5d^10^6s6d	^3^D_1_	[5]
1200	3021.499751(23)			33086.5108(3)	5d^10^6s6p	^3^P°_2_ – 5d^10^6s7d	^3^D_3_	[5]
300	3023.476256(27)	3023.476266(18)	−0.00001	33064.88229(20)	5d^10^6s6p	^3^P°_2_ – 5d^10^6s7d	^3^D_2_	[5]
30	3025.6080(5)	3025.60808(5)	−0.0001	33041.5861(5)	5d^10^6s6p	^3^P°_2_ – 5d^10^6s7d	^3^D_1_	[6]
60	3027.48949(5)	3027.489691(18)	−0.00020	33021.05129(20)	5d^10^6s6p	^3^P°_2_ – 5d^10^6s7d	^1^D_2_	[5]
4000	3125.670135(24)	3125.670105(15)	0.00003	31983.86780(15)	5d^10^6s6p	^3^P°_1_ – 5d^10^6s6d	^3^D_2_	[5]
3000	3131.551246(25)	3131.551249(17)	0.00000	31923.80347(18)	5d^10^6s6p	^3^P°_1_ – 5d^10^6s6d	^3^D_1_	[5]
4000	3131.842262(25)	3131.842247(15)	0.00002	31920.83735(16)	5d^10^6s6p	^3^P°_1_ – 5d^10^6s6d	^1^D_2_	[5]
700	3341.481483(22)	3341.481495(19)	−0.00001	29918.24299(17)	5d^10^6s6p	^3^P°_2_ – 5d^10^6s8s	^3^S_1_	[5]
9000	3650.156786(23)	3650.15679(2)	0.00000	27388.27973(17)	5d^10^6s6p	^3^P°_2_ – 5d^10^6s6d	^3^D_3_	[4]
3000	3654.839392(27)	3654.839367(19)	0.00003	27353.19079(15)	5d^10^6s6p	^3^P°_2_ – 5d^10^6s6d	^3^D_2_	[5]
500	3662.88282(4)	3662.88283(2)	−0.00001	27293.12646(18)	5d^10^6s6p	^3^P°_2_ – 5d^10^6s6d	^3^D_1_	[5]
2000	3663.280958(27)	3663.280953(20)	0.00000	27290.16034(15)	5d^10^6s6p	^3^P°_2_ – 5d^10^6s6d	^1^D_2_	[5]
50	3701.4324(6)	3701.4367(3)	−0.0043	27008.850(2)	5d^10^6s6p	^1^P°_1_ – 5d^10^6s9d	^3^D_2_	[6]^d^^e^
		3702.3437(4)		27002.234(3)	5d^10^6s6p	^1^P°_1_ – 5d^10^6s9d	^3^D_1_	
9	3704.1700(3)	3704.1700(3)	0.0000	26988.921(2)	5d^10^6s6p	^1^P°_1_ – 5d^10^6s9d	^1^D_2_	[6]^d^
50	3801.6603(4)			26296.831(2)	5d^10^6s6p	^1^P°_1_ – 5d^10^6s10s	^1^S_0_	[6]^d^
		3815.8254(4)		26199.214(2)	5d^10^6s6p	^1^P°_1_ – 5d^10^6s10s	^3^S_1_	
30	3901.8671(4)	3901.8674(2)	−0.0003	25621.4960(14)	5d^10^6s6p	^1^P°_1_ – 5d^10^6s8d	^3^D_2_	[6]^d^
		3903.6317(3)		25609.9161(17)	5d^10^6s6p	^1^P°_1_ – 5d^10^6s8d	^3^D_1_	
40	3906.37146(7)	3906.37149(7)	−0.00003	25591.9545(5)	5d^10^6s6p	^1^P°_1_ – 5d^10^6s8d	^1^D_2_	[5]
12000	4046.571500(25)	4046.57150(2)	0.00000	24705.29887(14)	5d^10^6s6p	^3^P°_0_ – 5d^10^6s7s	^3^S_1_	[4]
1000	4077.83806(3)	4077.83807(3)	−0.00001	24515.87647(18)	5d^10^6s6p	^3^P°_1_ – 5d^10^6s7s	^1^S_0_	[5]
70	4108.0577(3)	4108.0576(3)	0.0001	24335.5375(19)	5d^10^6s6p	^1^P°_1_ – 5d^10^6s9s	^1^S_0_	[6]^d^
		4140.05903(8)		24147.4347(5)	5d^10^6s6p	^1^P°_1_ – 5d^10^6s9s	^3^S_1_	
50	4339.22475(6)	4339.22466(4)	0.00009	23039.1145(2)	5d^10^6s6p	^1^P°_1_ – 5d^10^6s7d	^3^D_2_	[5]
		4343.61682(10)		23015.8183(5)	5d^10^6s6p	^1^P°_1_ – 5d^10^6s7d	^3^D_1_	
150	4347.495781(28)	4347.49575(3)	0.00003	22995.28349(14)	5d^10^6s6p	^1^P°_1_ – 5d^10^6s7d	^1^D_2_	[5]
12000	4358.337436(28)	4358.33745(2)	−0.00001	22938.08192(12)	5d^10^6s6p	^3^P°_1_ – 5d^10^6s7s	^3^S_1_	[4]
	4822.196(3)			20731.646(12)	5d^10^6s7s	^3^S_1_ – 5d^10^6s14p	^1^P°_1_	[7]
6	4882.991(3)	4882.9925(19)	−0.001	20473.527(8)	5d^10^6s7s	^3^S_1_ – 5d^10^6s13p	^1^P°_1_	[7]
	4896.861(3)			20415.544(12)	5d^10^6s7s	^3^S_1_ – 5d^10^6s13p	^3^P°_1_	[7]
20	4916.06952(6)	4916.06952(6)	0.00000	20335.7762(2)	5d^10^6s6p	^1^P°_1_ – 5d^10^6s8s	^1^S_0_	[5]
6	4970.368(3)	4970.3662(19)	0.002	20113.629(8)	5d^10^6s7s	^3^S_1_ – 5d^10^6s12p	^1^P°_1_	[7]
	4991.536(3)			20028.326(12)	5d^10^6s7s	^3^S_1_ – 5d^10^6s12p	^3^P°_1_	[7]
		5025.62491(6)		19892.4752(2)	5d^10^6s6p	^1^P°_1_ – 5d^10^6s8s	^3^S_1_	
30	5102.699(3)	5102.7017(19)	−0.003	19592.001(7)	5d^10^6s7s	^3^S_1_ – 5d^10^6s11p	^1^P°_1_	[7]
30	5137.930(3)			19457.670(11)	5d^10^6s7s	^3^S_1_ – 5d^10^6s11p	^3^P°_1_	[7]
		5219.429(3)		19153.852(12)	5d^10^6s7s	^1^S_0_ – 5d^10^6s14p	^1^P°_1_	
30	5290.729(3)	5290.728(2)	0.001	18895.733(8)	5d^10^6s7s	^1^S_0_ – 5d^10^6s13p	^1^P°_1_	[7]
6	5316.769(3)	5316.7713(19)	−0.002	18803.176(7)	5d^10^6s7s	^3^S_1_ – 5d^10^6s10p	^1^P°_1_	[7]
130	5354.029(3)			18672.329(9)	5d^10^6s7s	^3^S_1_ – 5d^10^6s10p	^3^P°_2_	[6]^d^
50	5384.615(3)			18566.267(10)	5d^10^6s7s	^3^S_1_ – 5d^10^6s10p	^3^P°_1_	[7]
	5393.454(3)	5393.456(2)	−0.002	18535.835(8)	5d^10^6s7s	^1^S_0_ – 5d^10^6s12p	^1^P°_1_	[7]
6000	5460.75312(4)	5460.75309(3)	0.00003	18307.40491(11)	5d^10^6s6p	^3^P°_2_ – 5d^10^6s7s	^3^S_1_	[4]
50	5549.635(3)	5549.633(2)	0.002	18014.207(7)	5d^10^6s7s	^1^S_0_ – 5d^10^6s11p	^1^P°_1_	[7]
600	5675.9229(6)	5675.9235(5)	−0.0006	17613.3909(15)	5d^10^6s7s	^3^S_1_ – 5d^10^6s9p	^1^P°_1_	[6]^d^
1000	5769.59838(7)	5769.59855(5)	−0.00017	17327.42299(16)	5d^10^6s6p	^1^P°_1_ – 5d^10^6s6d	^3^D_2_	[5]
30		5789.66824(8)		17267.3587(2)	5d^10^6s6p	^1^P°_1_ – 5d^10^6s6d	^3^D_1_	
900	5790.66289(7)	5790.66294(5)	−0.00005	17264.39254(16)	5d^10^6s6p	^1^P°_1_ – 5d^10^6s6d	^1^D_2_	[5]
400	5803.779(3)	5803.777(2)	0.002	17225.382(7)	5d^10^6s7s	^1^S_0_ – 5d^10^6s10p	^1^P°_1_	[7]
130	5859.245(3)			17062.316(8)	5d^10^6s7s	^3^S_1_ – 5d^10^6s9p	^3^P°_1_	[7]
30	6072.7132(12)	6072.7125(7)	0.0007	16462.5481(18)	5d^10^6s7s	^3^S_1_ – 5d^9^6s^2^(^2^D_5/2_)6p	^2^[3/2]°_1_	[6]^d^
50	6234.4024(9)	6234.4014(6)	0.0010	16035.5963(15)	5d^10^6s7s	^1^S_0_ – 5d^10^6s9p	^1^P°_1_	[6]^d^
600	6716.4293(10)	6716.4297(8)	−0.0004	14884.7535(8)	5d^10^6s7s	^1^S_0_ – 5d^9^6s^2^(^2^D_5/2_)6p	^2^[3/2]°_1_	[6]^d^
	6888.576(3)	6888.5708(18)	0.005	14512.795(4)	5d^10^6s7s	^3^S_1_ – 5d^10^6s8p	^1^P°_1_	[7]
1000	6907.4616(8)			14473.1055(17)	5d^10^6s7s	^3^S_1_ – 5d^10^6s8p	^3^P°_2_	[6]^d^
1000	7081.900(3)			14116.612(6)	5d^10^6s7s	^3^S_1_ – 5d^10^6s8p	^3^P°_1_	[7]
		7674.12(2)		13027.23(4)	5d^9^6s^2^(^2^D_5/2_)6p	^2^[3/2]°_2_ – 5d^10^6s10d	^3^D_3_	
30	7728.831(3)	7728.835(2)	−0.004	12935.001(4)	5d^10^6s7s	^1^S_0_ – 5d^10^6s8p	^1^P°_1_	[7]
		7973.041(4)		12538.818(6)	5d^10^6s7s	^1^S_0_ – 5d^10^6s8p	^3^P°_1_	
		7978.678(3)		12529.958(5)	5d^9^6s^2^(^2^D_5/2_)6p	^2^[3/2]°_2_ – 5d^10^6s11s	^3^S_1_	
		8181.6289(16)		12219.146(2)	5d^9^6s^2^(^2^D_5/2_)6p	^2^[3/2]°_2_ – 5d^10^6s7f	^3^F°_3_	
		8195.276(3)		12198.798(5)	5d^9^6s^2^(^2^D_5/2_)6p	^2^[3/2]°_2_ – 5d^10^6s9d	^3^D_3_	
		8200.2948(18)		12191.332(3)	5d^9^6s^2^(^2^D_5/2_)6p	^2^[3/2]°_2_ – 5d^10^6s9d	^3^D_2_	
		8204.7474(19)		12184.716(3)	5d^9^6s^2^(^2^D_5/2_)6p	^2^[3/2]°_2_ – 5d^10^6s9d	^3^D_1_	
		8505.140(3)		11754.367(5)	5d^10^6s7p	^3^P°_1_ – 5d^10^6s11s	^3^S_1_	
		8746.640(9)		11429.823(12)	5d^10^6s6d	^3^D_1_ – 5d^10^6s13p	^3^P°_1_	
		8757.429(2)		11415.741(3)	5d^10^6s7p	^3^P°_1_ – 5d^10^6s9d	^3^D_2_	
		8762.508(2)		11409.125(3)	5d^10^6s7p	^3^P°_1_ – 5^10^6s9d	^3^D_1_	
		8772.7443(19)		11395.812(3)	5d^10^6s7p	^3^P°_1_ – 5d^10^6s9d	^1^D_2_	
		8783.625(2)		11381.696(3)	5d^9^6s^2^(^2^D_5/2_)6p	^2^[3/2]°_2_ – 5d^10^6s10s	^3^S_1_	
		9050.918(10)		11045.571(12)	5d^10^6s6d	^1^D_2_ – 5d^10^6s12p	^3^P°_1_	
		9242.7827(9)		10816.2843(11)	5d^9^6s^2^(^2^D_5/2_)6p	^2^[3/2]°_2_ – 5d^10^6s8d	^3^D_3_	
		9253.3113(15)		10803.9774(18)	5d^9^6s^2^(^2^D_5/2_)6p	^2^[3/2]°_2_ – 5d^10^6s8d	^3^D_2_	
		9263.2398(16)		10792.3975(19)	5d^9^6s^2^(^2^D_5/2_)6p	^2^[3/2]°_2_ – 5d^10^6s8d	^3^D_1_	
		9278.6823(9)		10774.4359(11)	5d^9^6s^2^(^2^D_5/2_)6p	^2^[3/2]°_2_ – 5d^10^6s8d	^1^D_2_	
		9337.90(3)		10706.10(4)	5d^10^6s7p	^3^P°_2_ – 5d^10^6s10d	^3^D_3_	
	9425.934(13)	9425.946(2)	−0.012	10606.105(3)	5d^10^6s7p	^3^P°_1_ – 5d^10^6s10s	^3^S_1_	[7]^d^
		9543.999(10)		10474.915(11)	5d^10^6s6d	^1^D_2_ – 5d^10^6s11p	^3^P°_1_	
		9546.702(10)		10471.949(11)	5d^10^6s6d	^3^D_1_ – 5d^10^6s11p	^3^P°_1_	
		9601.776(10)		10411.884(11)	5d^10^6s6d	^3^D_2_ – 5d^10^6s11p	^3^P°_1_	
		9792.753(4)		10208.834(5)	5d^10^6s7p	^3^P°_2_ – 5d^10^6s11s	^3^S_1_	
	9968.955(13)	9968.9606(18)	−0.006	10028.3866(18)	5d^10^6s7p	^3^P°_1_ – 5d^10^6s8d	^3^D_2_	[7]^d^
		9980.4853(19)		10016.8067(19)	5d^10^6s7p	^3^P°_1_ – 5d^10^6s8d	^3^D_1_	
	9998.413(13)	9998.4139(11)	−0.001	9998.8451(11)	5d^10^6s7p	^3^P°_1_ – 5d^10^6s8d	^1^D_2_	[7]^d^
		10100.260(2)		9898.022(2)	5d^10^6s7p	^3^P°_2_ – 5d^10^6s7f	^3^F°_3_	
		10121.067(5)		9877.674(4)	5d^10^6s7p	^3^P°_2_ – 5d^10^6s9d	^3^D_3_	
		10128.722(3)		9870.208(3)	5d^10^6s7p	^3^P°_2_ – 5d^10^6s9d	^3^D_2_	
1600	10139.7937(8)	10139.7930(2)	0.0007	9859.4317(2)	5d^10^6s6p	^1^P°_1_ – 5d^10^6s7s	^1^S_0_	[11]^d^
		10180.073(7)		9820.421(7)	5d^10^6s6d	^1^D_2_ – 5d^10^6s10p	^1^P°_1_	
		10219.485(3)		9782.548(3)	5d^10^6s7p	^1^P°_1_ – 5d^10^6s9d	^3^D_2_	
	10240.342(13)	10240.347(3)	−0.005	9762.618(3)	5d^10^6s7p	^1^P°_1_ – 5d^10^6s9d	^1^D_2_	[7]^d^
		10296.644(2)		9709.241(2)	5d^10^6s6d	^3^D_2_ – 5d^10^6s7f	^3^F°_3_	
		10320.703(9)		9686.608(9)	5d^10^6s6d	^3^D_1_ – 5d^10^6s10p	^3^P°_2_	
		10333.991(2)		9674.152(2)	5d^10^6s6d	^3^D_3_ – 5d^10^6s7f	^3^F°_3_	
		10423.091(9)		9591.455(9)	5d^10^6s6d	^3^D_3_ – 5d^10^6s10p	^3^P°_2_	
		10431.730(11)		9583.512(10)	5d^10^6s6d	^1^D_2_ – 5d^10^6s10p	^3^P°_1_	
		10434.959(11)		9580.546(10)	5d^10^6s6d	^3^D_1_ – 5d^10^6s10p	^3^P°_1_	
		10500.793(11)		9520.482(10)	5d^10^6s6d	^3^D_2_ – 5d^10^6s10p	^3^P°_1_	
		10715.2745(13)		9329.9161(11)	5d^9^6s^2^(^2^D_5/2_)6p	^2^[3/2]°_2_ – 5d^10^6s9s	^3^S_1_	
		11021.698(3)		9070.528(3)	5d^10^6s7p	^1^P°_1_ – 5d^10^6s10s	^1^S_0_	
		11033.809(3)		9060.572(3)	5d^10^6s7p	^3^P°_2_ –5d^10^6s10s	^3^S_1_	
	11176.811(13)	11176.7868(16)	0.024	8944.6659(13)	5d^10^6s7s	^3^S_1_ – 5d^10^6s7p	^1^P°_1_	[7]^d^
1000	11287.4072(9)			8857.0053(7)	5d^10^6s7s	^3^S_1_ – 5d^10^6s7p	^3^P°_2_	[11]^d^
		11354.453(16)		8804.706(12)	5d^10^6s8s	^3^S_1_ –5d^10^6s13p	^3^P°_1_	
		11435.339(3)		8742.428(2)	5d^10^6s7p	^3^P°_1_ – 5d^10^6s9s	^1^S_0_	
		11686.7936(15)		8554.3253(11)	5d^10^6s7p	^3^P°_1_ – 5d^10^6s9s	^3^S_1_	
		11768.1869(11)		8495.1604(8)	5d^10^6s7p	^3^P°_2_ – 5d^10^6s8d	^3^D_3_	
		11950.3710(19)		8365.6515(13)	5d^10^6s7p	^1^P°_1_ – 5d^10^6s8d	^1^D_2_	
	12071.6031(21)	12071.6037(3)	−0.001	8281.63711(18)	5d^10^6s6p	^1^P°_1_ – 5d^10^6s7s	^3^S_1_	[9]^d^
		12127.8564(15)		8243.2244(10)	5d^9^6s^2^(^2^D_5/2_)6p	^2^[3/2]°_2_ – 5d^10^6s7d	^3^D_3_	
		12159.7612(15)		8221.5959(10)	5d^9^6s^2^(^2^D_5/2_)6p	^2^[3/2]°_2_ – 5d^10^6s7d	^3^D_2_	
		12378.069(13)		8076.595(8)	5d^10^6s6d	^3^D_1_ – 5d^10^6s9p	^3^P°_1_	
400		13426.3488(19)		7446.0051(10)	5d^10^6s7p	^3^P°_1_ – 5d^10^6s7d	^3^D_2_	
130		13468.487(2)		7422.7089(11)	5d^10^6s7p	^3^P°_1_ – 5d^10^6s7d	^3^D_1_	
		13479.286(3)		7416.7622(18)	5d^10^6s6d	^3^D_2_ – 5d^9^6s^2^(^2^D_5/2_)6p	^2^[3/2]°_1_	
200		13505.8513(19)		7402.1741(10)	5d^10^6s7p	^3^P°_1_ – 5d^10^6s7d	^1^D_2_	
200	13570.5721(23)	13570.573(2)	−0.001	7366.8713(12)	5d^10^6s7s	^1^S_0_ – 5d^10^6s7p	^1^P°_1_	[9]^d^
300	13673.3970(19)	13673.3972(19)	−0.0002	7311.4723(10)	5d^10^6s7s	^3^S_1_ – 5d^10^6s7p	^3^P°_1_	[9]^d^
		14062.368(5)		7109.235(2)	5d^10^6s7p	^1^P°_1_ – 5d^10^6s9s	^1^S_0_	
		14157.444(17)		7061.491(9)	5d^10^6s8s	^3^S_1_ – 5d^10^6s10p	^3^P°_2_	
600	15295.9752(23)	15295.975(2)	0.000	6535.8815(10)	5d^10^6s7s	^3^S_1_ – 5d^9^6s^2^(^2^D_5/2_)6p	^2^[3/2]°_2_	[9]^d^
300		16881.289(2)		5922.1006(8)	5d^10^6s7p	^3^P°_2_ – 5d^10^6s7d	^3^D_3_	
1600	16920.664(3)			5908.3197(10)	5d^10^6s6d	^1^D_2_ – 5d^10^6s5f	^1^F°_3_	[8]^d^
		16933.967(3)		5903.6784(10)	5d^10^6s6d	^1^D_2_ – 5d^10^6s5f	^3^F°_2_	
50	16942.479(3)			5900.7122(10)	5d^10^6s6d	^3^D_1_ – 5d^10^6s5f	^3^F°_2_	[8]^d^
		17010.329(3)		5877.1758(9)	5d^10^6s7p	^3^P°_2_ – 5d^10^6s7d	^3^D_1_	
80	17073.125(3)			5855.5592(10)	5d^10^6s6d	^3^D_3_ – 5d^10^6s5f	^3^F°_4_	[8]^d^
50	17109.898(3)			5842.9743(10)	5d^10^6s6d	^3^D_2_ – 5d^10^6s5f	^3^F°_3_	[8]^d^
30		17116.713(3)		5840.6479(10)	5d^10^6s6d	^3^D_2_ – 5d^10^6s5f	^3^F°_2_	
		17198.682(4)		5812.8115(13)	5d^10^6s7p	^1^P°_1_ – 5d1^0^6s7d	^3^D_2_	
		17206.411(3)		5810.2004(10)	5d^10^6s6d	^3^D_3_ – 5d1^0^6s5f	^1^F°_3_	
30		17213.269(3)		5807.8854(10)	5d^10^6s6d	^3^D_3_ – 5d^10^6s5f	^3^F°_3_	
		17267.887(4)		5789.5153(13)	5d^10^6s7p	^1^P°_1_ – 5d^10^6s7d	^3^D_1_	
160		17329.352(4)		5768.9805(13)	5d^10^6s7p	^1^P°_1_ – 5d^10^6s7d	^1^D_2_	
50		17436.051(3)		5733.6777(10)	5d^10^6s7s	^1^S_0_ – 5d^10^6s7p	^3^P°_1_	
		17983.121(5)		5559.2518(15)	5d^10^6s8s	^1^S_0_ – 5d^10^6s9p	^1^P°_1_	
		18078.115(12)		5530.040(4)	5d^10^6s6d	^1^D_2_ – 5d^10^6s8p	^1^P°_1_	
100		18131.7(5)		5513.69(14)	5d^10^6s6d	^3^D_3_ – 5d^9^6s^2^(^2^D_5/2_)6p	^2^[7/2]°_4_	
		18540.138(6)		5392.2307(17)	5d^10^6s6d	^3^D_3_ – 5d^10^6s8p	^3^P°_2_	
		19484.47(2)		5130.891(6)	5d^10^6s6d	^3^D_1_ – 5d^10^6s8p	^3^P°_1_	
70		19699.224(4)		5074.9566(10)	5d^9^6s^2^(^2^D_5/2_)6p	^2^[3/2]°_2_ – 5d^10^6s8s	^3^S_1_	
		21085.184(5)		4742.6668(11)	5d^10^6s7p	^3^P°_1_ – 5d^10^6s8s	^1^S_0_	
30		23259.244(6)		4299.3658(10)	5d^10^6s7p	^3^P°_1_ – 5d^10^6s8s	^3^S_1_	
		32159.788(13)		3109.4732(13)	5d^10^6s7p	^3^P°_1_ – 5d^10^6s8s	^1^S_0_	
200	35227.1(16)			2838.72(13)	5d^9^6s^2^(^2^D_5/2_)6p	[7/2]°_4_ – 5d^10^6s5g	^3^G_5_	[13]
		36313.026(10)		2753.8328(7)	5d^10^6s7p	^3^P°_2_ – 5d^10^6s8s	^3^S_1_	
600	39265.4(8)			2546.77(5)	5d^10^6s5f	^3^F°_2_ – 5d^10^6s5g	^3^G_3_	[13]
5000	39292.6(8)	39292.834(16)	−0.2	2544.9933(10)	5d^9^6s^2^(^2^D_5/2_)6p	^2^[3/2]°_2_ – 5d^10^6s6d	^3^D_3_	[13]
1500	39300.8(8)			2544.48(5)	5d^10^6s5f	^3^F°_3_ – 5d^10^6s5g	^3^G_4_	[13]
1000	39335.4(8)			2542.24(5)	5d^10^6s5f	^1^F°_3_ – 5d^10^6s5g	^1^G_4_	[13]
700	40050.4(8)			2496.86(5)	5d^10^6s5f	^3^F°_4_ – 5d^10^6s5g	^3^G_5_	[13]
		58648.59(6)		1705.0707(18)	5d^10^6s7d	^3^D_2_ – 5d^9^6s^2^(^2^D_5/2_)6p	[3/2]°_1_	
		64888.51(18)		1541.105(4)	5d^10^6s8p	^1^P°_1_ – 5d^10^6s9s	^1^S_0_	

a The observed intensities are quoted from the NIST compilation [3]. They are on the same scale, except for the lines quoted from Humphreys and Paul [13].

b The observed and Ritz wavelengths between 2000 Å and 20000 Å are given in standard air, otherwise in vacuum. Conversion from vacuum to standard air and vice versa was made by means of the five-parameter formula for the refractive index of air from Peck and Reeder [19]. The given uncertainties do not account for the uncertainties of the conversion formula.

c The Ritz wavelengths and their uncertainties are obtained in the least-squares level optimization procedure by means of the computer code LOPT [28]. They are applicable to emission of electrodeless discharge lamps with Ar buffer gas pressure of 33 Pa. The value in this column is blank for the lines that alone determine one of the energy levels involved in the transition.

d The original measurements reported in these sources were adjusted in the present work (see text).

e This line was excluded from the level optimization procedure (see Sec. 2.2).

## Footnotes

1 All uncertainties given in the present paper are meant to be on the level of one standard deviation (or, equivalently, at the 68 % confidence level).

2 Conversion from vacuum to standard air and vice versa was made in the present paper using the five-parameter formula for the refractive index of air from Peck and Reeder [19].

3 Kaufman [15] did not specify the frequency of the exciting radiation.

4 We assumed that the Ar pressure in the lamps used by Terrien {28} was equal to the nominal pressure before the lamps were sealed (see next paragraph).
